# Enhanced Learning through Multimodal Training: Evidence from a Comprehensive Cognitive, Physical Fitness, and Neuroscience Intervention

**DOI:** 10.1038/s41598-017-06237-5

**Published:** 2017-07-19

**Authors:** N. Ward, E. Paul, P. Watson, G. E. Cooke, C. H. Hillman, N. J. Cohen, A. F. Kramer, A. K. Barbey

**Affiliations:** 1 Beckman Institute for Advanced Science and Technology, University of Illinois at Urbana-Champaign, Urbana, IL United States of America; 2 Department of Psychology, Tufts University, Medford, MA United States of America; 3 Department of Kinesiology and Community Health, University of Illinois at Urbana-Champaign, Urbana, IL United States of America; 4 Department of Psychology, University of Illinois at Urbana-Champaign, Champaign, IL United States of America; 5 Neuroscience Program, University of Illinois at Urbana-Champaign, Champaign, IL United States of America; 6 Department of Internal Medicine, University of Illinois at Urbana-Champaign, Champaign, IL United States of America; 7 Department of Bioengineering, University of Illinois at Urbana-Champaign, Champaign, IL United States of America; 8 Department of Speech and Hearing Science, University of Illinois at Urbana-Champaign, Champaign, IL United States of America; 9 Carle R. Woese Institute for Genomic Biology, University of Illinois at Urbana-Champaign, Champaign, IL United States of America; 10 Department of Psychology, Northeastern University, Boston, MA United States of America

## Abstract

The potential impact of brain training methods for enhancing human cognition in healthy and clinical populations has motivated increasing public interest and scientific scrutiny. At issue is the merits of intervention modalities, such as computer-based cognitive training, physical exercise training, and non-invasive brain stimulation, and whether such interventions synergistically enhance cognition. To investigate this issue, we conducted a comprehensive 4-month randomized controlled trial in which 318 healthy, young adults were enrolled in one of five interventions: (1) Computer-based cognitive training on six adaptive tests of executive function; (2) Cognitive and physical exercise training; (3) Cognitive training combined with non-invasive brain stimulation and physical exercise training; (4) Active control training in adaptive visual search and change detection tasks; and (5) Passive control. Our findings demonstrate that multimodal training significantly enhanced learning (relative to computer-based cognitive training alone) and provided an effective method to promote skill learning across multiple cognitive domains, spanning executive functions, working memory, and planning and problem solving. These results help to establish the beneficial effects of multimodal intervention and identify key areas for future research in the continued effort to improve human cognition.

## Introduction

A longstanding goal of research in the psychological and brain sciences is to enhance brain health and to deliver long lasting cognitive benefits that improve the quality of human judgment and reasoning in complex, real-world environments. A central question in this effort is whether the exclusive focus on computer-based cognitive training in science and industry is warranted^1–7^. A largely unexplored possibility is that skill learning is enhanced by the addition of intervention modalities that are designed to promote neuroplasticity and engender changes in brain structure and function that promote learning^8, 9^.

Several recent multimodal training endeavors have shown promise in positively changing cognition when supplementing cognitive training with physical exercise^8, 10^. For example, Fabre and colleagues^11^ found that a group that received combined cognitive training and physical exercise outperformed other unimodal training groups (exercise-only and cognitive training-only) on memory tasks, such as a story memory task and a paired associates task. Likewise, Oswald and colleagues^12^ found that individuals randomly assigned to a combined exercise and cognitive training group showed large improvements in a composite measure of cognitive abilities (i.e., processing speed, memory, attention, reasoning) after 30 training sessions compared to exercise-only and cognitive training-only groups, and these improvements were still evident up to five years later compared to individuals assigned to cognitive training or exercise in isolation. While the underlying mechanisms for these synergistic effects are not fully understood, researchers have suggested that exercise may aid learning via long-term potentiation and hippocampal neurogenesis as well as a variety of other structural and functional brain changes^13–16^, which might then “prime” the brain for subsequent enhancement via cognitive training^17, 18^. If this is true, then one might expect performance on theoretically motivated cognitive games, such as the ones in the current study, to be enhanced when paired with exercise compared to cognitive games that have not been supplemented with exercise.

An emerging area of research in cognitive neuroscience investigates the combined effects of cognitive training and non-invasive brain stimulation (via transcranial direct current stimulation or tDCS). Many argue that active (or anodal) tDCS ultimately influences cognition by increasing cortical excitability^19^. For example, Martin and colleagues^20^ found that individuals in an active stimulation group demonstrated better performance on attention and working memory tasks after 10 sessions of dual n-back training compared to those in a sham control group due to increased cortical excitability produced by the application of tDCS. Similarly, Ditye and colleagues^21^ found that individuals receiving active tDCS along with cognitive training outperformed individuals in a cognitive training-only group on measures of inhibition. To explain these results on a mechanistic level, some have argued that pairing tDCS with cognitive training can enhance plasticity and strengthen task related cortical networks, which has the potential to generate behavioral improvements on trained and possibly even untrained tasks^22, 23^.

The widespread advocacy and adoption of brain training software in the public eye – and what we perceive as an opportunity to strengthen the effects of computer-based training through the addition of multiple intervention modalities^24, 25^ – motivate a careful investigation of the evidential basis of brain training methods and an examination of the benefits of multimodal interventions to enhance new skill learning.

### Overview

Our investigation examines whether contemporary approaches to brain training – including computer-based cognitive training, physical exercise training, and non-invasive brain stimulation – enhance learning of trained tasks and improve performance on untrained tests of intellectual ability within healthy, young adults. Our cognitive training protocol incorporates several training paradigms from cognitive psychology that have shown promise for enhancing executive functions^5, 26–31^ and is implemented within a software package that is adaptive (user-dependent and dynamic task difficulty), novel (training of unfamiliar tasks), engaging (interesting and enjoyable), and comprehensive (designed to improve critical building blocks of cognition relevant to a broad array of tasks and skills). In addition to investigating the effects of a 16-week cognitive training intervention (Games group; *n* = 59), we examined multimodal approaches to brain training, including a physical exercise group (Exercise and Games: EG group; *n* = 68) and a group that engaged in physical exercise and then received brain stimulation during cognitive training (Exercise, Stimulation, and Games: ESG group; *n* = 61). Prior research has shown that physical exercise and brain stimulation can enhance learning and memory, in both children and older adults, and therefore may amplify the effects of brain training in our population of healthy, young adults^21, 22, 32, 33^.

## Methods

The University of Illinois at Urbana-Champaign Institutional Review Board approved all procedures used (IRB14212), and all methods were performed in accordance with the relevant guidelines and regulations. Five hundred and eighteen participants were recruited from the local community and randomly assigned to one of five groups after giving informed written consent. Four of the five groups involved either a unimodal or multimodal training regimen that lasted 16 weeks with each week consisting of three, 70 minute sessions (i.e., 48 sessions or 210 minutes of training per week). The Games group engaged in six computerized training games based on executive function and working memory tasks for all 48 sessions. The EG group engaged in physical exercise for 28 sessions as well as computerized training games for 20 sessions. The ESG group also engaged in 28 sessions of physical exercise similar to the EG group; however, they further received active HD-tDCS during their 20 sessions of computerized training games. The Active Control group (AC) engaged in different computerized training games based on visual search and change detection for the same duration and frequency as the Games group (i.e., 48 sessions over 16 weeks). Game order was randomized at each session for all computerized training. Each game was played for approximately 10 minutes on tablets in the laboratory each session, and games in both the experimental and active control groups were designed to be adaptive both within and across sessions. Finally, there was a no contact control group (Passive Control or PC) that completed pre- and post-tests but did not engage in training. In addition, all groups completed a battery of untrained transfer tasks prior to and following the training period. On the basis of prior cognitive training research, the battery of tasks measured multiple cognitive abilities, including measures of executive function, working memory, episodic memory, and fluid intelligence. In addition to this battery of tasks, aerobic fitness was measured at pre-test and post-test for all participants using a graded exercise test designed to measure maximal oxygen consumption (VO_2_ max).

### Participants

Of the 518 participants enrolled in the study, 318 finished. Subject attrition was not related to condition, *χ* ^*2*^(4, *n* = 518) = 2.85, *p* = 0.58, nor were there group differences in gender (*χ* ^*2*^(4, *n* = 318) = 7.86, *p* = 0.10), age (*F*(4, 313) = 1.87, *p* = 0.12), or education (*X* ^2^(4) = 1.88, *p* = 0.76).

### Conditions

Participants in the Games group (27 female|32 male; average age = 24) performed six games all based on psychological tasks of executive function and working memory. These games were created as part of a cognitive training package in which the component tasks (games) were chosen because they have shown promise in engendering training and transfer effects in the extant literature^5^, though this is the first instance of them being used in a multimodal setting. Five of the six games were identical to those used in previous research^5^, and one of the six games was new to this study (i.e., Ante Up, which was based on measures of mental planning).

Participants in the EG group (29 female|39 male; average age = 25) were enrolled in a physical exercise intervention administered in a group setting of up to 20 participants per class. Participants in each class were divided into groups of 5 or fewer participants per trainer. During the exercise sessions, heart rate was monitored using a Polar heart rate monitor^34^. Participants completed a total of 28 exercise sessions (12 the 1^st^ month, eight the 2^nd^ month, four the 3^rd^ month, and four the 4^th^ month). The warm-up included dynamic stretching and light activity to prepare the body for exercise. There was a walk/run portion that varied in time and distance among the sessions. In addition, there was a high intensity cardiovascular and resistance training (HICRT) protocol that was split into two segments of three sets of three-to-four exercises. Participants completed one- to two-minutes of jump rope and a four-minute power series between the two segments. The HICRT exercises varied among sessions and included exercises involving bodyweight, resistance bands, kettlebells, body bars, and suspension training. There was also a drills-and-skills portion that varied among sessions and included whole-body training with equipment such as battle ropes, sand bags, ladders, medicine balls, and parachutes. Every exercise session finished with dynamic stretching and a cool-down routine. In addition to the physical exercise sessions, participants in the EG group completed 20 sessions of the same computerized training games as the Games group. These game sessions started the 2^nd^ month with four sessions and then increased to eight sessions per month in the 3^rd^ and 4^th^ months. During the game sessions, participants received sham tDCS.

Participants in the ESG group (40 female|21 male; average age = 24) engaged in the same 28 group exercise sessions, but rather than sham stimulation, they received active tDCS. This involved “high-definition” electrode montages (HD-tDCS) to precisely target brain regions by sending a weak current from two small electrodes (anodes) on the left and right side of the front of the head to two receiving electrodes (cathodes) on the back of the head. Electrode sites were prepared with highly conductive gel. Stimulation was applied via a modified Soterix Medical 1 × 1 Transcranial Direct Current Low-Intensity Stimulator (Model 1300 A) to deliver the current to two sites. For both groups, anodal electrodes were placed over left and right dorsolateral prefrontal cortex (F3 and F4) while the cathodal electrodes were placed over the left and right occipital cortex (O1 and O2) according to international 10–20 standards^35^. Participants experienced a short exposure period at the beginning of each session to acclimate to the stimulation sensation before the longer stimulation period began. Participants in the ESG group received a total of 2.0 mA current over the entire scalp, split between each anode resulting in roughly 1.0 mA to each hemisphere. Stimulation ramped up and ramped down over 30 seconds at the beginning and end of each session, with continuous current applied over the course of 30 minutes. Participants in the EG group underwent all of the same procedures as those in the ESG group except that the current lasted only 30 seconds at the start and end of the session. The comparable ramp-up/ramp-down sequence for the EG group was used to elicit the same ‘tingling’ sensation and to balance expectations about whether or not stimulation had been applied^36^.

Participants in the AC group (32 female|34 male; average age = 25) performed three versions of a visual search task and three versions of a change detection task. In prior research, training on these tasks has not led to changes on executive function, working memory, episodic memory, or fluid intelligence; however, participants did show substantial enhancements in visual search and change detection performance^6, 7^. The AC group followed the same training schedule as the Games group. Participants in the PC group (32 female|32 male; average age = 27) completed the same pre/post tasks at the beginning of the study and then again after 16 weeks; however, they had no contact with any of the research team during the 16-week intervention period.

### Assessments

Participants completed a battery of tasks both before and after the training intervention. On the basis of prior cognitive training research, the battery of tasks measured multiple cognitive abilities, including measures of executive function, working memory, episodic memory, and fluid intelligence. At the latent level, construct scores were extracted via confirmatory factor analysis (CFA) models that specified *a priori* the three variables in each construct (EF: Garavan^37^, Keep Track^38^, Stroop^39^; WM: Read Span^40^, Rotation Span^41^, Symmetry Span^42^; EM: IFR Words^7^, IFR Pictures^43^, Paired Associates^44^; GF: BOMAT^45^, Number Series^46^, Letter Sets^47^). This process was repeated at post-test, and both pre- and post-test models converged with acceptable fit indices (Pre: RMSEA = 0.06, CFI = 0.91, AIC = 187.71; Post: RMSEA = 0.04, CFI = 0.98, AIC = 153.91). Factor score estimates were extracted and analyzed using IBM SPSS AMOS 22^48^.

In addition to this battery of tasks, aerobic fitness was measured at pre-test and post-test for all participants using a graded exercise test designed to measure maximal oxygen consumption (VO_2_ max). An ANOVA using the VO_2_ max scores yielded a significant Group by Assessment interaction, *F*(4,305) = 11.46, *p* < 0.001, *η*_*p*_^*2*^ = 0.13. Pairwise comparisons indicated that only the two groups that engaged in physical exercise (EG and ESG) significantly increased their cardiorespiratory fitness levels, though they did not significantly differ from each other.

### Expectations

At the end of the intervention, participants reported how much their participation in the study had changed their abilities. The 14 abilities (e.g., overall intelligence, divided attention, long term memory, emotional regulation, etc.) were rated on a scale from 1 (dramatically worse) to 7 (dramatically better) and were aggregated into a composite expectation score. The training and transfer results did not differ when this expectation score was included as a continuous covariate.

## Results

### Training Performance

To examine the time-course of cognitive training, we calculated standardized improvement scores at each of the 20 training sessions by converting the average difficulty for each task to a standardized score for each of the four active training groups (three experimental groups and an active control group). We then subtracted first session performance from subsequent sessions for each participant^7^. We conducted separate repeated measures analyses of variance on these standardized scores for each training group as a function of training session and found that all training groups (including the active control group) significantly improved over the course of study (Supplemental Table 1). The active control group (AC; *n* = 66) received training on visual search and change detection tasks that were not designed to transfer to tests of executive function (EF), working memory (WM), episodic memory (EM), or fluid intelligence (GF) that were administered before and after the interventions. The passive control (PC; *n* = 64) group did not engage in any training but completed the same transfer tasks.

**Figure 1:**
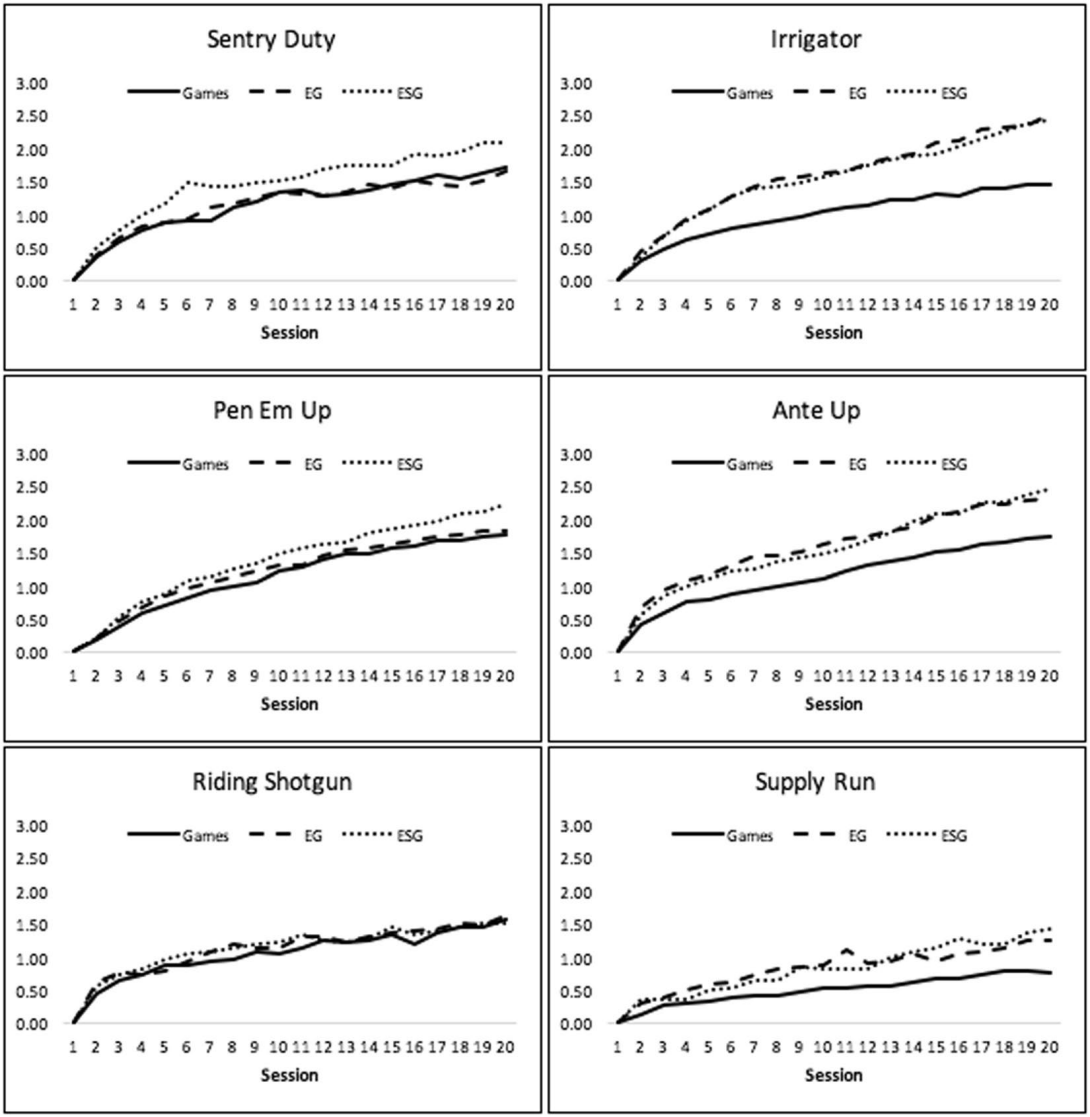
Enhanced Training Performance Over Time. Standardized improvement units are on y-axis. There was a significant group difference in terms of slope and area under the curve for all games except Riding Shotgun (alpha = 0.05). Games = computerized game training. EG = Exercise and computerized game training. ESG = Exercise, brain stimulation, and computerized game training.

**Figure 2:**
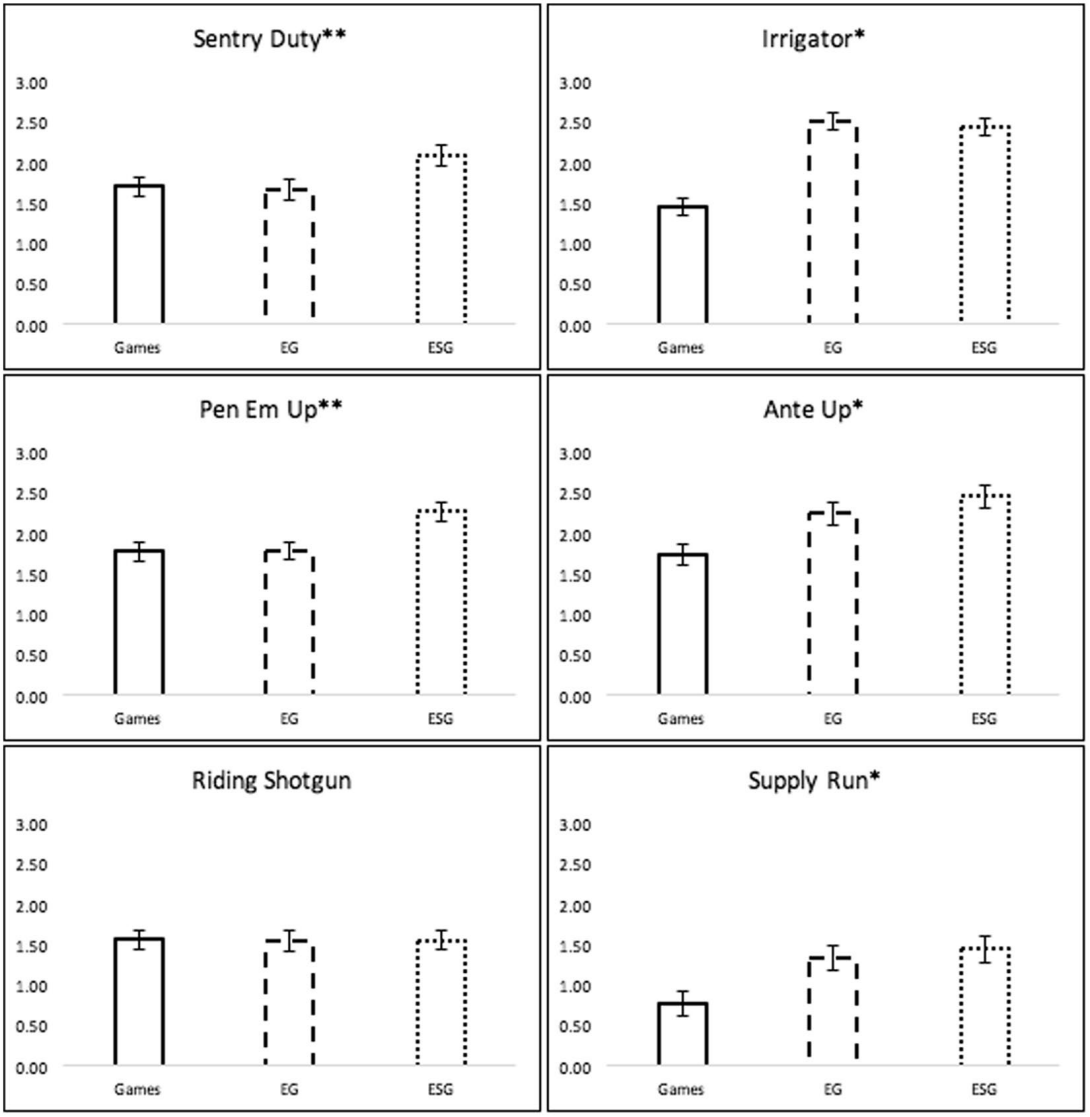
Enhanced Training Performance at Session 20. Standardized improvement units on y-axis. **Indicates ESG significantly greater than EG and Games (alpha = 0.05); *indicates both ESG and EG significantly greater than Games (alpha = 0.05); no significant effect of group at session 20 for Riding Shotgun. Error bars represent +/− one standard error of the mean.

To examine the relative benefits of multimodal approaches to brain training, we compared standardized training curves across 20 sessions for each of the experimental groups (Fig. 1). Next, we compared these groups in terms of their performance at session 20 for each of the cognitive training tasks and found an effect of Group for all but one of the tasks (Supplemental Table 2). Planned pairwise comparisons indicated that both multimodal groups (ESG and EG) achieved significantly greater performance compared to the unimodal (Games) group on Irrigator (based on visuospatial reasoning), Ante Up (based on mental planning), and Supply Run (based on working memory updating). Interestingly, the ESG group achieved significantly greater performance than the Games and EG group on Sentry Duty (based on dual n-back) and Pen Em Up (based on task switching (Fig. 2).

#### Transfer Performance

In addition to testing for enhancement of learning as a result of multimodal training, analyses were conducted to explore whether enhanced training performance led to enhanced performance on untrained pre- and post-intervention assessment tasks. A significant effect of Group at post-test while covarying pre-test performance would serve as evidence of transfer^7^. Comparable post-intervention performance was observed across the unimodal and multimodal intervention groups for tests of general intelligence (examined for latent constructs of executive function, working memory, episodic memory, and fluid intelligence). In each case, evidence for significant transfer to these constructs was not obtained. At the individual task level, one of the twelve tasks demonstrated a significant effect of Group at post-test while controlling for pre-test performance (Table 1). However, pairwise comparisons revealed that this effect represents superior performance in the ESG group relative to the passive (rather than active) control group (*p* < 0.05). These findings provide clear evidence for the strengths but also the limitations of a subset of contemporary brain training methods – establishing the beneficial effects of multimodal training on learning while also identifying the need for effective methods to engender transfer to general facets of intellectual ability.

**Table 1:** ANCOVA Results for Constructs and Individual Transfer Tasks.

Task	Group		
	F	p	η_p_^2^
EF	1.91	0.11	0.02
Garavan	0.75	0.56	0.01
Keep Track	3.30	*0.01*	0.04
Stroop	0.58	0.68	*0.01*
WM	0.88	0.48	0.01
Read Span	1.16	0.33	0.02
Rotation Span	1.28	0.28	0.02
Symmetry Span	0.34	0.85	0.00
EM	0.94	0.44	0.01
IFR Words	0.80	0.53	0.01
IFR Pictures	1.15	0.34	0.02
Paired Associates	0.38	0.83	0.01
GF	0.38	0.82	0.01
BOMAT	0.44	0.78	0.01
Number Series	0.65	0.63	0.01
Letter Sets	0.35	0.84	0.00

Note: Values in italics indicate significant values (alpha = 0.05). EF = Executive Function. WM = Working Memory. EM = Episodic Memory. GF = Fluid Intelligence. IFR = Immediate Free Recall.

## Discussion

We have conducted one of the largest and most comprehensive randomized brain training studies performed to date and provided novel evidence that a multimodal intervention – based on cognitive training, brain stimulation, and exercise training – can enhance learning (i.e., performance during cognitive training). Specifically, game performance was enhanced in five of the six games when exercise and/or exercise and brain stimulation was included compared to training that was bound to a single intervention modality. For three of the games (Irrigator, Ante Up, and Supply Run), exercise enhanced learning in terms of training game performance compared to the unimodal group. This extends previous training research that has found exercise-related enhancements on learning^18^, though to our knowledge this is the first study to show these enhancements in young adults using executive function and working memory training tasks.

In addition to the learning enhancements from exercise, adding brain stimulation (as well as exercise) to the intervention led to enhanced training performance on two of the games (Sentry Duty and Pen Em Up) compared to the other multimodal group and the unimodal group. Previous research with healthy younger adults has found that pairing active brain stimulation with cognitive tasks can enhance performance^49^. The current research extends these findings to multimodal interventions, demonstrating enhanced learning on executive function and working memory games using a combination of exercise and brain stimulation.

Our experiment was designed to provide a rigorous investigation of the efficacy of multimodal approaches to brain training in healthy, young adults. Specifically, we applied random assignment to groups, investigated a large sample (*n* = 318), implemented 20–48 training sessions over 16 weeks, applied multiple measures per cognitive construct, utilized both active and passive control groups^50, 51^, and examined the role of expectancies. Our findings therefore inform scientific and public policy recommendations about the multimodal interventions investigated in the present study. Indeed, a key set of issues in the design of brain training methods pertains to the specific cognitive skills that are trained (e.g., executive functions) and the length of the intervention protocol administered (e.g., 210 minutes per week for 16 weeks). It remains possible, for example, that transfer effects may depend on a different cognitive training protocol (not based on executive function) and training for a longer duration (more than 20 sessions). Participants may need greater exposure to each of the training games to engender transfer to general intelligence (but see ref. 52). We believe these alternatives should be examined in future research given that the majority of studies reporting beneficial effects of cognitive training are consistent with the administered protocol (i.e., with a focus on executive functions and training that is less than 20 sessions^27, 53^).

There are several other limitations that should be considered when evaluating multimodal training effects. For instance, given the high level of attrition in the current study, it is possible that those who dropped might have been less motivated or had different baseline abilities compared to those who completed the study. If that were the case, then perhaps those individuals would have more to gain from multimodal training efforts. For those who did complete the study, we observed real-time multimodal training effects but only weak evidence for transfer. We used one program of exercise and one type of brain stimulation for our multimodal intervention to try to maximize our potential for observing transfer, but it is possible that a different type of exercise program and brain stimulation setup might lead to greater evidence of transfer. Indeed, most of the research on the efficacy of fitness training for cognition and brain health comes from exercise programs that are focused on improvements in cardiorespiratory fitness rather than mixed programs of resistance, skills, and cardiorespiratory fitness training as used in the present study^15, 54^. Also, despite our large sample size and random assignment to training group, it is possible that individual differences in cognitive abilities obscured any potential transfer effects.

While previous research has found robust transfer effects^26, 27^, this has not always been the case^4, 7^. Furthermore, the current study differs from several of the previous studies in that our focus on was the effects of multimodal (vs. unimodal) training. We also included a battery of training tasks rather than a single task, and at least for our measures of fluid intelligence we used the BOMAT whereas some previous studies have used other matrix reasoning tasks. Given the wide variety of training and transfer results in the literature and the lack of multimodal interventions, further replication and studies of the size, scope, and duration of the present randomized controlled trial are needed.

The importance of establishing methods to enhance cognitive and brain health has been increasingly recognized in the psychological and brain sciences, but the efficacy of state-of-the-art brain training methods remains the subject of continued research and debate. Our study provides evidence that the administered multimodal training protocol significantly enhances learning across multiple cognitive domains, spanning executive functions, working memory, and planning and problem solving. Our findings therefore motivate further investigation of how best to optimize multimodal interventions based on subject-specific tailoring (e.g., to establish the nature and duration of training based on cognitive and brain imaging phenotypes of learning) and to advance the scientific effort to improve general facets of intellectual ability.

## Electronic supplementary material

Supplemental Information
