# Supplementary material for: Enhanced Learning through Multimodal Training: Evidence from a Comprehensive Cognitive, Physical Fitness, and Neuroscience Intervention
Source: Sci Rep. 2017 Jul 19;7:5808. doi: 10.1038/s41598-017-06237-5 (PMC5517605; doi:10.1038/s41598-017-06237-5)
Supplement: Supplementary file 1 — Supplemental Information [file 41598_2017_6237_MOESM1_ESM.pdf]

Supplemental Information

Enhanced Learning through Multimodal Training: Evidence from a Comprehensive Cognitive, Physical Fitness, and Neuroscience Intervention

N. Ward[1,2*], E. Paul[1], P. Watson[1], G. E. Cooke[1], C. H. Hillman[1,3,4,5,6,10], N. J. Cohen[1,4,5], A. F. Kramer[1,4,5], and A. K. Barbey[1,4,5,6,7,8,9*]

[1]Beckman Institute for Advanced Science and Technology, University of Illinois at Urbana-Champaign, Urbana, IL, United States of America

[2]Department of Psychology, Tufts University, Medford, MA, United States of America

[3]Department of Kinesiology and Community Health, University of Illinois at Urbana-Champaign, Urbana, IL, United States of America

[4]Department of Psychology, University of Illinois at Urbana-Champaign, Champaign, IL, United States of America

[5]Neuroscience Program, University of Illinois at Urbana-Champaign, Champaign, IL, United States of America

[6]Department of Internal Medicine, University of Illinois at Urbana-Champaign, Champaign, IL, United States of America

[7]Department of Bioengineering, University of Illinois at Urbana-Champaign, Champaign, IL, United States of America

[8]Department of Speech and Hearing Science, University of Illinois at Urbana-Champaign, Champaign, IL, United States of America

[9]Carle R. Woese Institute for Genomic Biology, University of Illinois at Urbana-Champaign, Champaign, IL, United States of America

[10]Department of Psychology, Northeastern University, Boston, MA, United States of America.

*Corresponding authors
Nathan Ward; E-mail: nathan.ward@tufts.edu (NW)
Aron K. Barbey; E-mail: barbey@illinois.edu (AKB)
Decision Neuroscience Laboratory
Beckman Institute for Advanced Science and Technology
University of Illinois at Urbana-Champaign
405 North Mathews Avenue, Urbana, IL, 61801
Web: http://decisionneurosciencelab.org/

Table 1. Training Performance Collapsed Across All Tasks.

Training group	*F*	*P*	η_p^2
Games	273.36	*<0.001*	0.83
EG	213.56	*<0.001*	0.78
ESG	227.88	*<0.001*	0.80
AC	527.94	*<0.001*	0.89

Note: Values in italics indicate significant values (alpha = 0.05). Games = computerized game training. EG = Exercise and computerized game training. ESG = Exercise, brain stimulation, and computerized game training. AC = Active control (i.e., training on visual search and change detection, which served as a control condition for the other three training groups).

Table 2. Group Differences in Training Performance at Session 20.

	Group		
Training game (Ability)	*F*	*p*	η_p^2
Sentry Duty (Dual N-Back)	3.61	*0.03*	0.04
Irrigator (Visuospatial Reasoning)	33.89	*<0.001*	0.28
Pen Em Up (Task Switching)	6.36	*<0.001*	0.07
Ante Up (Mental Planning)	7.14	*<0.001*	0.08
Riding Shotgun (Visuospatial Working Memory)	0.01	0.99	0.00
Supply Run (Working Memory Updating)	5.25	*0.01*	0.06

Note: Values in italics indicate significant values (alpha = 0.05).

Table 3b. Descriptive Statistics for Transfer Measures

	Pre					*Post*				
Task	Games	EG	ESG	AC	PC	Games	EG	ESG	AC	PC
EF										
Garavan (errors)	3.45 (4.84)	5.04 (15.00)	2.80 (3.91)	2.80 (4.08)	3.35 (4.71)	3.63 (4.81)	2.72 (4.27)	3.15 (4.27)	2.54 (4.79)	3.41 (4.80)
Keep Track (items recalled)	38.59 (7.37)	*39.34 (5.49)*	39.03 (6.12)	39.38 (6.48)	38.69 (5.61)	40.61 (8.01)	40.41 (7.99)	42.22 (6.80)	40.71 (8.12)	37.64 (7.88)
Stroop (cost RT)	120.06 (97.67)	103.64 (87.31)	73.16 (80.38)	108.53 (86.08)	109.81 (90.68)	79.09 (82.05)	83.41 (61.96)	70.29 (75.06)	75.38 (73.34)	85.58 (72.96)
WM										
Read Span (total score)	16.97 (9.26)	18.49 (9.20)	19.56 (9.19)	19.62 (8.35)	18.88 (8.55)	17.37 (9.68)	17.34 (10.08)	19.36 (9.39)	19.56 (8.33)	16.67 (9.12)
Rotation Span (total score)	11.47 (6.38)	11.12 (6.61)	13.63 (6.63)	13.09 (5.93)	12.38 (5.75)	11.42 (6.35)	12.25 (6.33)	13.45 (7.64)	13.35 (6.12)	11.16 (5.85)
Symmetry Span (total score)	18.34 (7.87)	17.39 (7.79)	19.11 (8.37)	19.14 (7.26)	16.91 (7.44)	19.12 (8.89)	17.67 (7.12)	19.69 (8.91)	18.86 (7.07)	17.44 (8.02)
EM										
IFR Words (items recalled)	23.25 (7.05)	22.04 (5.91)	23.97 (5.77)	23.39 (5.24)	23.64 (6.41)	24.59 (6.43)	24.76 (5.89)	25.51 (6.32)	23.89 (6.12)	24.84 (6.60)
IFR Pictures (items recalled)	26.32 (5.27)	25.69 (5.39)	26.13 (4.91)	25.48 (5.11)	26.36 (5.00)	27.46 (6.03)	26.67 (5.03)	27.87 (6.30)	26.36 (5.66)	26.48 (4.86)
Paired Associates (items recalled)	4.19 (2.29)	3.78 (2.49)	3.59 (2.15)	3.56 (2.33)	4.02 (2.38)	4.10 (2.57)	4.18 (2.39)	3.95 (2.44)	3.92 (2.28)	4.41 (2.38)
GF										
BOMAT (correct trials)	5.9 (1.71)	5.75 (2.23)	6.39 (1.84)	5.66 (1.83)	5.45 (1.77)	5.98 (1.92)	6.06 (2.09)	6.67 (2.16)	6.14 (2.21)	5.91 (2.26)
Number Series (correct trials)	6.97 (2.12)	6.85 (2.21)	7.47 (2.14)	6.58 (1.95)	6.81 (2.00)	7.25 (2.06)	7.20 (1.96)	7.00 (2.31)	6.71 (2.11)	6.89 (2.02)
Letter Sets (correct trials)	10.97 (1.92)	11.15 (1.91)	11.35 (1.89)	10.82 (2.29)	10.88 (2.04)	10.83 (2.55)	11.01 (2.31)	11.42 (2.32)	11.05 (2.31)	10.86 (2.51)

Note: Values represent Means (SD). Games = computerized game training. EG = Exercise and computerized game training. ESG = Exercise, brain stimulation, and computerized game training. AC = Active control. PC = Passive control.
